# Caregivers perception of common neonatal illnesses and their management among rural dwellers in Enugu state, Nigeria: a qualitative study

**DOI:** 10.1186/s12889-023-15582-2

**Published:** 2023-04-11

**Authors:** Onyinye H Chime, Chizoma I. Eneh, Isaac N Asinobi, Uchenna Ekwochi, Ikenna Kingsley Ndu, Obinna C Nduagubam, Ogechukwu F Amadi, Donatus Chidiebere Osuorah

**Affiliations:** 1 Department of Community Medicine, Enugu State University of Science and Technology, Enugu State University Teaching Hospital (ESUT-TH) Parklane, PMB 1030, Enugu, Enugu State Nigeria; 2 Department of Paediatrics, Enugu State University of Science and Technology and Enugu State University Teaching Hospital (ESUT-TH) Parklane, PMB 1030, Enugu State, Enugu, Enugu State Nigeria; 3 Child Survival Unit, Medical Research Council Unit, UK, Fajara, Serrakumda The Gambia

**Keywords:** Caregiver, Causes, Management, Neonatal illnesses, Perception, Rural communities

## Abstract

**Background:**

Neonatal mortality continues to be a challenge in Nigeria, where low-quality care, caregivers’ ignorance of signs of neonatal illnesses, and prevalent use of unorthodox alternatives to health care predominate. Misconceptions originating and propagating as traditional practices and concepts can be linked to adverse neonatal outcomes and increased neonatal mortality. This study explores the perceptions of causes and management of neonatal illness among caregivers in rural communities in Enugu state, Nigeria.

**Methods:**

This was a cross-sectional qualitative study among female caregivers of children residing in rural communities in Enugu state. A total of six focus group discussions (FGDs) were conducted; three in each of the communities, using an FGD guide developed by the researchers. Using pre-determined themes, thematic content analysis was used to analyze the data.

**Results:**

The mean age of respondents was 37.2 ± 13.5 years. Neonatal illnesses were reportedly presented in two forms; mild and severe forms. The common causes of the mild illnesses reported were fever, jaundice, eye discharge, skin disorders, and depressed fontanelle. The severe ones were convulsion, breathlessness/difficulty or fast breathing, draining pus from the umbilicus, and failure-to-thrive. The caregivers’ perceptions of causes and management of each illness varied. While some believed these illnesses could be managed with unorthodox treatments, others perceived the need to visit health centers for medical care.

**Conclusions:**

Caregivers’ perception on the causes and management of common neonatal illnesses in these communities is poor. Obvious gaps were identified in this study. There is a need to design appropriate interventions to dispel the myths and improve the knowledge of these caregivers on neonatal illnesses towards adopting good health-seeking behaviours.

## Background

The neonatal period refers to the first 28 days after birth. Broadly, neonatal illnesses may be defined as the disturbances of the body’s normal state and abnormal function of a baby before 28 days of age. These illnesses may develop while the fetus is growing in-utero, during labor and delivery, or after birth. Commonly encountered neonatal illnesses include prematurity, neonatal infections, low blood sugar, respiratory dysfunction, hemolytic disorders of the newborn, birth trauma, and congenital malformations. These manifest as fevers, breathing difficulties, jaundice, convulsions, eye discharge, skin changes, and umbilical anomalies. Most neonatal illnesses if not appropriately treated or treatment is delayed; could lead to mortality.

Neonatal mortality has remained a global health concern [1]. The World Health Organization (WHO) reports that nearly half (47%) of children worldwide who die before the age of five are neonates [2]. Within the first month of life, 2.4 million children died in 2020 – approximately 6,500 neonatal deaths every day – with about a third of these deaths occurring within the first day after birth and close to three-quarters occurring within the first week of life [3]. Approximately 99% of these deaths occur in developing countries [1].

Efforts have been made globally to reduce neonatal mortality. Among the 17 Sustainable Development Goals (SDGs) set by United Nations in 2015, the 3rd goal (target 2) aims at achieving a reduction in neonatal mortality rate (NMR) to no more than 12 deaths per 1000 live births, thereby putting a stop to millions of avoidable newborn deaths by 2030 [4]. However, the global decline in NMR is slower in Nigeria and other sub-Saharan African countries, too slow to anticipate the actualization of the SDG 3 goal and target for the majority of low-income countries [5]. In Nigeria, NMR dropped from 72 deaths per 1000 live births in 2010 to 37 in 2015 and 35 deaths per 1000 live births in 2020 (a mere 1.7% drop over five years) [3]. This is much less than that of Kenya that decreased by more than a half from 47 to 22 per 1000 live births over a five year period (a 4% decline in over a 5-year duration) [6].

The neonatal period remains the most critical period for an infant’s survival [3]. In sub-Saharan Africa, the major causes of neonatal mortality include infections (sepsis, pneumonia, diarrhea), birth asphyxia, and prematurity [7]. Neonatal mortality continues to be a challenge in Nigeria as a result of poor antenatal care, unhygienic delivery practices with adverse delivery and perinatal events, poor breastfeeding practices, poor maternal knowledge as well as delays in recognition of signs of illness in babies [8–10]. In Nigeria, NMR is higher in children born to mothers in rural communities [11]. According to a 2017 study that used data from the 2013 Nigeria Demographic and Health Survey (NDHS); NMR for rural and urban populations was 36 and 28 deaths per 1000 live births, respectively [12]. Risk factors identified in this study included lack of electricity access, small birth size (as a proxy for low birth weight), male gender and birth interval < 2 years. Furthermore from the NDHS of 2018 there is a wide difference in the level of education of mother in the rural and urban areas in Nigeria. While urban women have completed a median of 11 years of education, their counterparts in rural communities have completed zero median years [11]. Additionally, women in rural areas have higher fertility rates and poor access to antenatal care from skilled birth attendants than those in urban areas. Although rural households outnumber urban ones, 42% of households in rural communities still depend on non-portable sources of drinking water, and about 33% have no household toilet facility [11].

A study carried out several years ago in the study settings recorded poor knowledge of most of the danger signs of neonatal illness amongst mothers and caregivers [13]. In other rural settings, cultural beliefs and practices relating to newborn care have been reported [14, 15]. Based on these studies, traditional medicines and home remedies were primarily used to manage neonatal illnesses. In some cases, traditional practices prevent caregivers from taking the newborns outside their homes even if they are sick [14]. Orthodox healthcare is sought following referrals from traditional doctors or when the baby is perceived to be in a critical condition. These misconceptions originating from traditional concepts of these conditions and their management are directly linked to poor knowledge and utilization of healthcare services for newborns. These ‘old wives tales’ though lacking in merit, are passed from one generation to another. If not correctly identified and documented, some of these beliefs could negatively influence caregivers’ communication and adoption of any scientific medical intervention.

Misconceptions originating and being propagated as traditional practices and concepts can be linked to adverse neonatal outcomes and increased neonatal mortality. While the focus of other studies has been on ascertaining the caregivers’ knowledge level, there is a need for more data on the myths and perception of neonatal illnesses and the management of such within their locality. Therefore, this study explored caregivers’ perceptions of the causes and management of neonatal illness in rural communities in South East Nigeria. Findings from this study will provide insight into their understanding of neonatal illnesses, putting into context their cultural beliefs and other socioeconomic factors. Furthermore, it will form a baseline for further studies, aiding the design of appropriate community-based interventions to dispel myths and improve caregivers’ knowledge of neonatal illnesses for early and prompt treatment.

## Methods

### Study area

The study was conducted in Enugu state, one of Nigeria’s five states in the Southeast geopolitical zone. Enugu state has seventeen local government areas of which three are urban, three are semi-urban, and eleven are rural areas.

This was a community-based study conducted in two rural communities in Nkanu East local government area with a population of about 169,703 indigenes and a landmass of 795 square kilometers [16]. These communities were selected based on findings of poor knowledge of danger signs and health-seeking practices in newborns by caregivers reported in these communities in a previous study by some of the author of this article [13]. However the participants were not the patients of the authors. Inhabitants of these communities, Ndiuno Uwani Akpugo and Nomeh Unateze, are of the Igbo ethnic nationality and predominantly subsistence farmers and petty traders with low level of education (largely primary education). Located in each community is one primary health care facility which is meant to operate 24 h daily but only operate during the day due to factors such as insecurity and epileptic power supply. These facilities are manned mainly by community health extension workers. Their main source of drinking water is their local streams which occasionally dry out during dry seasons. Although the NMR in the state as at 2018 was 21 deaths per thousand live births, this value is higher in rural communities such as the study area compared to urban communities [11].

### Study period

This study was conducted from March to May 2022. This included activities carried out for community entry, meeting with selected community women to aid in the study and the data collection in the two communities.

### Study design and population

This was a cross-sectional, descriptive, qualitative study among female caregivers of children residing in rural communities in Enugu state. Recruited for the study were direct female caregivers of children under five years of age (mothers, grandmothers, or aunts) who were permanent residents in the selected communities, with a history of caring for neonates presently or having cared for neonates within the last five years.

### Data collection

A total of six focus group discussions (FGDs) were conducted; three in each of the communities. Each FGD comprising about 8 to 12 women lasted between 45 and 60 min. A total of 58 women participated in the study. Using this number of FGDs, a saturation point where no new information was obtained was quickly reached. The researchers developed the FGD guide using questions adapted from a study on knowledge of newborns’ danger signs and health-seeking practices of mothers and caregivers [13]. Using open-ended questions, FGD was used to obtain an in-depth view of caregivers’ perception of causes and management of neonatal illnesses in their communities. This tool was pre-tested among caregivers of children attending children’s outpatient clinics at Enugu State University Teaching Hospital.

The FGDs were conducted in the local dialect (Igbo) at central and non-threatening locations (school, town hall, and church hall). Women leaders in both communities were engaged to facilitate access to the study participants within the communities with shared experiences of nursing neonates. To gain a broad range of perspectives and insights on this research, participants from different age groups able to shed light on this topic were purposively selected. Data was collected by a team of researchers made up of eight doctors. During the recruitment phase, adequate and accurate information about the study was provided to the potential participants and their written consents were obtained. This process was repeated before commencement of the discussions to re-confirm the decision of the caregivers to participate in the study. The FGDs for each community were conducted on non-market days. The interviews were held at various times on the same day to ensure the women’s optimal participation.

### Data entry, processing and analysis

Data was recorded electronically using an audio recorder and hand written notes. Thematic content analysis was used to analyze the data. The recorded discussions and notes were first translated into English by experts with good command of both languages. Next, the researchers compared the scripts with the written notes for precision and quality assurance. Finally, using the hybrid coding approach, the transcripts were coded by a statistical analyst manually line-by-line using ‘comments’ in Microsoft Word. Codes were assigned to recurring words and phrases in the text. The codes identified common neonatal illnesses prevalent in the communities. These were further categorized into mild and severe categories. A second round of coding was done to describe the various excerpts of the pre-determined themes; causes of neonatal illnesses and its management. The audio recordings and transcripts are safely stored in the library of the Department of Pediatrics. Data will be destroyed once it is no longer needed after two years.

## Results

A total of six FGDs were conducted using the FGD guide. Table 1 shows the socio-demographic profile of the participants. The mean age of the participants was 37.2 ± 13.5 years, and 48.3% of the participants had completed their primary education. Subsistence farming (51.7%) and petty trading (29.3%) were their principal occupations, as they relied on their farm produce for sustenance. (Table 1)

**Table 1:** Socio-demographic profile of the participants

Variable	Number (n = 58)	Percentage (%)
**Age (years) categorized**		
Mean ± SD	37.2 ± 13.5	
< 20 years	2	3.4
20–29 years	17	29.3
30–39 years	22	37.9
≥ 40 years	17	29.3
**Relationship of respondent**		
Mother	36	62.1
Grandmother	11	19.0
Others/Caregiver	11	19.0
**Highest Educational Qualification**		
None	6	10.3
Completed primary	28	48.3
Completed junior secondary	11	19.0
Completed senior secondary	10	17.2
Completed tertiary	3	5.2
**Occupation**		
Unemployed	5	8.6
Farming	30	51.7
Trading	17	29.3
Tailoring	3	5.2
Other businesses	1	1.7
Salary earner	2	3.4

### Causes of neonatal illnesses

Several causes of neonatal illnesses were reported by participants in this study; fever, vomiting, crying excessively, refusal of feeds (breast milk or any other food item), jaundice, convulsion, etc. However, the participants reported that not all these were common in the neonatal period. They opined that neonatal illnesses could present in mild or severe forms. The common mild illnesses reported were fever, jaundice, eye discharge, skin disorders, and depressed fontanelle. The severe ones were convulsion, breathlessness/difficulty or fast breathing, draining pus from the umbilicus, and failure-to-thrive. Most participants believed that a caregiver will almost always know when a neonate is ill and that once treated, the baby usually responds favourably.

### Caregiver perception of common causes of neonatal illnesses

#### Mild

##### Fever

This is presumed to be a common illness in neonates. In most cases, the caregivers reported it occurs together with crying excessively, refusal of feeds, and irritability. For neonates, a maternal condition during birth plays a role in neonatal fever. One caregiver recalls her ordeal,

*I had fever during the last days before I put to bed. My baby started having fever few days after birth. My mother said I transferred the fever to my baby when he was in the womb*.

Another caregiver opined that,

*Neonates that underwent stress during labour (prolonged labour) or assisted delivery occasionally come down with fever. This fever resolves after some days without any medication*.

##### Jaundice

Popularly called ‘*iba ocha na anya*” (yellowness of the eyes). Jaundice typically occurs in neonates born to mothers with febrile illness during pregnancy and also in premature babies. For the majority of the caregivers,

*jaundice is a common presentation and usually nothing to worry about as it resolves within some days*.

However, some disagreed with this. One of the caregivers said,

*jaundice should not be toyed with. It can cause convulsion in children and in severe cases, death*.

##### Eye discharge

The caregivers know that a child with discharge from the eyes is unwell. One of the caregivers opined that eye discharge results from neonatal jaundice. Another caregiver corroborated this view as she recalled her experience a few days after the birth of one of her children,

*when my child presented with eye discharge, I was advised to expose the child under the sun and the eye discharge stopped after some days.*

Another caregiver suggested that eye discharge could result from applying a local eyeliner, ‘*otanjele*,‘ on the child. According to her, eyeliner is applied to help the baby have clear and bright eyes all his/her life. Also, it is used for beautification as it makes the eyes of the baby look sharp.

##### Skin disorders

Two common skin presentations reported in newborns were skin rashes and discoloration. Body rashes in children commonly appear within one week after birth and are popularly known as ‘*oku rice’*; these rashes resemble burns sustained from boiling rice hence its name. The other symptom, hypo-pigmentation, is known as ‘*nlacha*’ (seborrheic dermatitis). It presents first on the face and chest and occasionally spreads to other body parts. An elderly caregiver reported thus,

*most children are born with ‘nlacha’. Whatever causes it is domicile in their mothers’ wombs. There is nothing much you can do to prevent this in these children*.

##### Depressed fontanelle (*Ntiwa isi*)

Another presentation is ‘*ntiwa isi’* (sunken or depressed anterior or posterior fontanelle). For the caregivers,

*there is something breathing inside the head which is another sign of illness in the baby. Babies with such depressions are irritable, lose weight and have slow developments*.

The participants believe this is not a regular occurrence and wondered why only children born to mothers in rural areas have this opening on their skulls. One young mother said,

“*This illness occurs if the mother is not adequately hydrated during pregnancy*. *Because we in the rural areas do not have access to good water like those in the cities, this may be why it occurs more here*.

For another caregiver,

*This could also be a sign that the child’s head is not well formed*.

#### Severe

##### Convulsion

Most participants opined that.

*convulsion in neonates results following untreated febrile conditions. In rare cases, there might not be any fever as the fever is said to be ‘hidden’ yet capable of triggering fits in these babies*.

Other caregivers opined that,

*convulsion is caused by evil spirits that come around to harm babies. This is why most episodes occur at night when the presence of these supernatural beings is vividly felt*.

##### Breathlessness

If a child is breathless, the child has caught a cold. It was generally agreed that the child had not been covered well by the mother. It usually presents together with cough in neonates. An elderly caregiver reported this;

*The young mothers especially do not take time to cover their newborns well. Because the mothers are usually hot, they expose themselves likewise their babies not knowing their body is not the same as that of the baby. These young ones then catch cold and become breathless*.

##### Umbilical redness or drainage of pus

It was generally reported that umbilical redness or drainage of pus is due to lack of care of the umbilical area, and as such, it begins to smell, draining the pus and the child can get febrile. In addition, a caregiver responded thus,

*neonates develop umbilical redness or pus draining from the umbilicus because they are circumcised without checking if they have jaundice as jaundice prevents healing of the umbilical cord.*

##### Failure to thrive (*Nta*)

Another neonatal illness is ‘nta’. For the caregivers, an infant with a normal appetite who, instead of gaining weight, continuously loses weight (failure to thrive) has ‘nta’. This failure to thrive, the caregivers say, is common in their communities and is easily identified by elderly mothers. A middle-aged caregiver reported that,

*This disorder is transmissible. Though there are no apparent signs or symptoms in adults, the mother can be a carrier, thereby infecting other children when she carries them*.

### Caregivers’ perception of management of common neonatal illnesses

#### Mild

The caregivers’ perceptions of management varied. It ranged from the use of local remedies to the use of orthodox medications. While some thought these illnesses could be managed with unorthodox treatments, others perceived the need to visit health centers for medical care.

##### Fever

For the caregivers, it is normal for a child to have a fever occasionally. If a child has a fever, a mother said.

*It is advisable to use cold water to clean the baby’s body and expose the baby adequately. This controls the fever in some cases*.

Most caregivers thought giving the febrile child ‘paracetamol’ (acetaminophen analgesia) would combat the fever. Another mother said,

*palm kernel oil known as ‘enuaku’ can be used to massage the body. It can also be given orally to the child say about a teaspoon. It helps combat fever in neonates*.

If the fever persists after the home remedies have been exhausted, it is generally recommended that the baby be taken to the health center.

##### Jaundice

For most rural dwellers, the yellowness of the eyes can be managed at home. One of the caregivers,

*Children with yellowness of the eyes are kept under the early morning sun; preferably before 10am daily till the yellowness of eyes clears. The child is also given glucose water and Abidec (a popular multivitamin) to clear the jaundice*.

However, if the symptom persists after a week, the caregivers echo that the child is taken to the hospital. Occasionally they take the children to the maternities where they were born for immediate treatment.

“*The nurses manage such cases but if severe, they know when to refer to health centers*. “

They all said they do not take jaundiced children to TBAs because, in their opinion, the TBAs are not competent enough to manage such cases;

“*no one wants to risk the life of her child*,” a mother reported. Furthermore, an elderly mother reported thus,

*When a child has jaundice, he/she should not be circumcised as it will make the jaundice linger more than expected. This can even lead to the death of the child*.

##### Eye discharge

To clear eye discharge in neonates,

“*the application of mother breast milk while breastfeeding the child*.

,“ was a popular opinion. Other recommendations include the application of fresh palm wine and sugar solution as eye drops. However, a young mother criticized this practice saying,

*these practices were condemned by health care workers during one of my visits to a health centre. My baby was placed on cod liver oil and Abidec (multivitamin syrup) which the healthcare worker claimed would keep the baby’s eyes cleared for life. My baby is still on these medications as at six months and she is yet to have any eye discharge*.

While several caregivers recommended the use of local eyeliner ‘otanjele’ for the management of abnormal eye discharge, a mother recounted her ordeal with its application,

“*In order to beautify my child with the local eyeliner as is popular in our setting, my child developed yellowish eye discharge which I attribute to how the ‘otanjele’ was applied to her eyes. Perhaps during application of the local eye liner, the eye was traumatized resulting in the discharge. I can’t really say what happened. All I know is that my child had difficulty opening one of her eyes with yellowish fluid coming out of it and I had to discontinue application of the eye liner*,” she said.

She recollected that the eye cleared without any intervention after three days.

##### Skin disorders

To manage body rashes, the caregivers popularly recommend that,

*mothers should use warm ‘rice water’ to bathe the child for about three days to one week*. *This normally clears the rashes within days; unless there are underlying illnesses the mother transmitted to the child while in the womb*.

Further probing reveals that ‘rice water’ is obtained after sieving boiled rice. Another mother resorted to orthodox practice,

*with recent developments in medicine, it is no longer trendy to use the ‘rice water’. The baby continues to have rice odour throughout that period which is not nice. There are ointments which also clear the rashes like ‘vista-plus’ (contains Clotrimazole, Beclomethasone dipropionate and Gentamicin), ‘nixoderm’(sulphur ointment) and ‘smuth cream’ (Calcium Dobesilate, Hydrocortisone, Lidocaine and Zinc).*

To treat seborrheic dermatitis ‘*nlacha*’, a caregiver recommends this concoction,

*“use local seeds known as ‘akiriso’ (Guizotia abyssinica). After grinding it, mix with ashes and potash. It clears the dermatitis within days.“*

##### Depressed fontanelle (*Ntiwa isi*)

Once this is observed, the child is taken to an herbalist who applies a semi-solid substance to cap the fontanelle. An elderly caregiver responds thus,

“*There is a dedicated herbalist who specialises in managing this in our setting. Not all herbalists know how to do this. The topical medication applied on the opening stops the breathing. This substance is expected to stick to the fontanelle till the ailment heals which can take weeks”.*

#### Severe

##### Convulsion

Convulsion was established to have different causes. The causative factor determines its management.

*“Those caused by evil spirits can be managed at home. To drive away these evil spirits, topical local ointments are effective. Highly recommended is ‘enuaku’ as the smell repulses evil spirits. To make this local ointment more effective, mix it together with ginger, garlic and dried ‘scent leaves’*,” was recommended by the caregivers.

Traditional healers make these local ointments, and the caregivers do not know what the significant constituents are. They, however, believe it is effective. To manage convulsions resulting from febrile illnesses, a caregiver responded thus,

“*first and foremost when a baby has fever, you have to apply the local ointments which reduces fever thereby preventing convulsion. Afterwards you can go ahead and find out the cause of the fever. These local ointments should be a household item for every woman within the childbearing age for emergency situations. They prevent and treat febrile convulsion in children*. *This practice is unknown to the orthodox health practitioners and is effective.*“

Once a caregiver notice twitches with fixation of eyes or convulsions in the child, she immerses the child in a basin of cold water. This act of water immersion is a common practice. A mother recommends that,

*You will have to immerse the child in cold water. Once the baby passes faeces while inside the water, it means the convulsion has subsided. After this, you will start applying ‘enuaku’ on the body of the child as it abates convulsion*.

Flames of fire over the feet of convulsing children have controlled the episodes in children. The majority of the women frowned at this practice. A caregiver recalled an incident that happened in the community years back;

“*a woman in a bid to manage neonatal convulsion placed the sole of the baby’s feet over flames of fire. Accidentally, the child while convulsing fell into the fire and died from the burns. After that incident, the use of flames of fire to manage convulsion in children though still practised, is discouraged*,” she said.

##### Breathlessness

To manage this condition, it was generally recommended that thicker clothing is used to cover the child. Other home remedies include massaging with a towel and hot water (hot enough for the mother to immerse her hand in) and applying Robb ointment, which warms up the baby and relaxes the fast breathing. For another caregiver, nothing works as fast as applying local ointment,

‘*if you want the child’s breathing to normalize as fast as possible, apply ‘enuaku’ to his/her feet and place over the flames for a while*,” she said.

##### Umbilical redness or drainage of pus

It was generally reported that this presentation could signify that the umbilicus is infected. The general opinion was that proper care of the umbilical stump would prevent this presentation. On means of caring for the umbilical region, a caregiver responded,

*you have to use hot water on the region while bathing the child morning and night. Clean with methylated sprit about three times daily and apply Vaseline (a popular brand of mineral oils and petroleum jelly) on it. Such care will ensure the umbilicus is clean and prevent poor healing of the umbilical cord.*

For another caregiver, adequate management of jaundice before circumcision is recommended to avert this condition. She said,

if jaundice is not well managed, it affects the entire body and results in poor healing of the umbilicus.

It was generally recommended that a child with an umbilicus that is healing poorly or draining pus should be taken to the health facility.

##### Failure to thrive (*Nta*)

Though the affected neonates may appear normal initially, older women claim to make an early diagnosis of the latent phase of the ailment. A grandmother recommends that special local herbs (leaves) are ground and applied to the child’s skin.

*“With daily application of this herb, goat-like hairs will be seen coming out the child’s body. These hairs reduce over the days. As this process is ongoing the weight of the child improves gradually till the hairs do not appear at all during application of the herbs. At that point, the child is healed.”* said the grandmother.

Furthermore, to prevent person-to-person transmission,

*the primary caregiver should also apply the herbs on her own body to ensure she doesn’t contract this ailment*.

There is no known orthodox management of this ailment. Therefore, herbal leaves are solely used in the management of this disorder.

## Discussion

This study sought to provide insight into caregivers’ understanding of neonatal illnesses, putting into context their cultural beliefs and other socioeconomic factors. The wide age range of participants captures the ages of caregivers usually involved in the care of neonates in our local environment. It represents a population that should be able to articulate clearly their knowledge of the subject matter. The majority of our participants attained only primary education and low maternal education has been linked to higher neonatal mortality and poor perception of common illnesses and their management in a number of studies [17, 18].

A simple categorization of severity for neonatal illnesses was used in this study. Various scoring systems have been devised that assess neonatal disease severity and outcome and are used to study trends across neonatal units [19, 20]. These, however, require investigations and are pretty complex. The Modified Sick Neonatal Score (MSNS) has been studied in rural and urban areas but also has parameters that participants in this study could not easily measure [20].

The participants in this study were able to identify neonatal illnesses as being either mild or severe. Similarly, in a study by Ekwochi et al. [13] in Enugu on mothers’ perception of neonatal illness and health-seeking behavior, all but one mother was aware of some signs in the newborn suggestive of imminent danger. Although fever and jaundice were perceived as features of mild illness in the present study, pathological jaundice however is seen as a danger sign in neonates while fever is not [21]. Bad experiences from previous illnesses have also been identified as a determinant of the severity assessment [8]. Several authors have documented the underestimation of signs of severe illness in neonates attributable to maternal educational level [8, 13, 17, 22, 23]. The findings in this study contradict those of several studies conducted in rural communities of Africa and Asia where fever and jaundice were seen as danger signs requiring immediate medical intervention [8, 14, 24]. Vomiting and frequent passage of loose stool were also perceived as uncommon features of neonatal illness in this study. Despite the fact that vomiting and diarrhea have been consistently reported as features of severe illness, they are, however not common in neonatal period as they are attributed to changes in feeds and are usually self-limiting [23, 25]. Thus the participants might not have perceived these presentations as a threat.

The signs of severe illness noted in this study were those listed in the 2021 NICE guideline listing signs and symptoms of severe illness in the postnatal period [26]. The World Health Organization (WHO) and United Nations Children’s Fund (UNICEF) [27] had earlier listed fast breathing, convulsions, and drainage of pus from the umbilicus among the nine danger signs signifying severe neonatal illness. The categorization of fast breathing/breathlessness and convulsion as signs of severe illness by participants of this study is likely due to these two events being often seen as ‘near death’ events and frightening to behold. Breathlessness signifies some degree of respiratory distress. Respiratory distress occurs in up to 7% of neonates and is the leading cause of mortality in early neonatal period (0–7 days) [28, 29]. Such babies are 2 to 4 times more likely to die than those without respiratory distress [30]. It is also the leading cause of morbidity and neonatal intensive care admissions, especially in developing countries [31].

Perceived causes of neonatal illness were diverse and illness specific. These, however, still fell into the two categories documented by Odwe et al. [32] Biomedical (natural) causes included poor hygienic practices (pus from the umbilical cord), maternal nutrition and disease (depressed anterior fontanelle and skin rash), and undue exposure to harsh environmental conditions (breathlessness). Other conditions, like convulsions, were attributed to evil spirits (traditional causes) [32].

Numerous studies have been carried out on the maternal perception of several neonatal illnesses, varying across different cultures and with different socioeconomic backgrounds.^14,33−35^ Caregivers in this study perceived jaundice as being caused by febrile illnesses and prematurity. In other parts of Nigeria, Africa, and Asia, jaundice has variously been attributed to mainly: mosquitoes and yellow fever (Lagos, Nigeria), eating palm oil, infections, a breach in food restrictions, lack of hygiene, and evil spirits [14, 33, 35]. The view of mothers in this study that jaundice was harmless and would clear on its own was in stark contrast to that of women in Accra, Ghana, and Nepal, most of whom saw it as being dangerous, requiring medical attention [34, 35].

Other conditions such as pus in the umbilicus, skin rash (nlacha), failure to thrive (nta), and depressed anterior fontanelle (ntiwa isi) were generally blamed on the mother, with causes ranging from poor hygiene, inadequate nutrition and hydration, to conditions inherent in the mother. This depicts the poor knowledge of causes of illnesses that occurs with poor educational attainment, especially in localities with inadequate training on causes of neonatal illnesses, as in our study [13]. The belief that convulsions are caused by evils spirit was entertained by most caregivers in this study. This is a common belief in various communities within and outside Nigeria and corroborates the traditional belief component of the etiology of neonatal illnesses, as noted in earlier studies [32, 33].

Participants in this study resorted to home remedies for most neonatal illnesses, including those perceived as severe. These were employed first, with hospital care only sought when symptoms persisted. This sequential health-seeking approach is deterioration from the findings of Ekwochi et al. in Enugu six years ago, where about 50% of mothers took their babies to the hospital immediately after signs of illness were noticed [13]. The reason for this is unclear. It however appears to be due to a mixture of worsening economic situation in the country, poor health seeking behaviour due to over reliance on home remedies as well as myths that are held tightly to despite efforts at impacting basic knowledge about neonatal illnesses. Observation similar to those obtained in this study has been made in different parts of the country and other developing countries for different neonatal illnesses [36–38]. In Zaria, northwestern Nigeria, Igboanusi and co-workers reported that only 16.9% of caregivers took their children to the hospital immediately after noticing jaundice [36]. Mothers in the Netrakona district of Bangladesh would only take their jaundiced children to the hospital on referral from the traditional healer [37]. In a survey carried out in six sub-Saharan African countries in 2017, it was observed that only in Nigeria and the Democratic Republic of Congo would a significant proportion of mothers take their children to the hospital following recognition of breathlessness in the child [38]. However, in countries like the Central African Republic, Chad, Malawi, and Sierra Leone, mothers would not seek medical attention for their children despite adequate recognition of signs of pneumonia [38]. Several reasons have been given for the use of home remedies. These include cultural beliefs, feared adverse outcomes in hospitals, belief in faith healers, lack of money, and lack of transport [13, 27].

This study is the first to draw attention to these life-threatening neonatal care practices in these communities. The finding has implications for both public and policy interventions. For the public, it provides a clear insight into the possible causes of neonatal deaths in the communities. Identifying these myths and misconceptions is the unique finding of this study. It brings to bear the need for urgent intervention by healthcare workers and opinion leaders to adopt behavioural change communication models to dispel unsafe cultural practices and beliefs. Emphasis should be on preventing delays in seeking appropriate health care for the sick neonate. Similar studies in India and Tanzania reported such delays resulting from harmful practices [14, 39]. However, an interventional study in Enugu, Nigeria, showed that educating mothers using the Integrated Management of Childhood Illnesses (IMCI) model improved mothers’ knowledge of neonatal illnesses and the need for appropriate and prompt care of their sick neonates [40]. The government should also design policies to promote available and affordable maternal and child health services and reward caregivers and babies who visit health facilities.

### Limitations of the study

The transcripts were not returned to all the participants.

## Conclusions

Caregivers’ perception on the causes and management of common neonatal illnesses in these communities is poor. Obvious gaps were identified in this study. Appropriate interventions involving evidence-based newborn care practices should be designed to dispel the myths and improve the knowledge of these caregivers on neonatal illnesses. These interventions should be socio-culturally contextualized, targeting high-risk neonatal-care practices and result in substantial behavioral change and modification in the community. This will ensure good health-seeking behaviours as well as early and prompt treatment of common neonatal illnesses.

## Data Availability

The datasets generated and analyzed during the current study are available from the corresponding author upon reasonable request.

## Abbreviations

FGD: Focus Group Discussions
WHO: The World Health Organization
SDGs: Sustainable Development Goals
NMR: Neonatal Mortality Rate
MSNS: The Modified Sick Neonatal Score
UNICEF: United Nations Children’s Fund
TETFUND: Tertiary Education Trust Fund
